# CCAAT enhancer binding protein delta activates vesicle associated membrane protein 3 transcription to enhance chemoresistance and extracellular PD-L1 expression in triple-negative breast cancer

**DOI:** 10.1186/s13046-024-03041-8

**Published:** 2024-04-16

**Authors:** Yan Zhao, Yangyang Yu, Xiangmin Li, Ayao Guo

**Affiliations:** 1 Department of Breast Surgery, The First Hospital of China Medical University, No. 155, Nanjing North Street, Heping District, Shenyang, Liaoning 110001 P.R. China; 2 Department of Radiation Oncology, The First Hospital of China Medical University, No. 155, Nanjing North Street, Heping District, Shenyang, Liaoning 110001 P.R. China; 3 Department of Oncology, Shengjing Hospital of China Medical University, No. 36, Sanhao Street, Heping District, Shenyang, Liaoning 110004 P.R. China

**Keywords:** CEBPD, VAMP3, Triple-negative breast cancer, Autophagy, Paclitaxel resistance, Immune evasion

## Abstract

**Background:**

Chemoresistance and immunosuppression are two major obstacles in the current anti-cancer treatments. This study investigates the involvements of a CCAAT enhancer binding protein delta (CEBPD)/vesicle associated membrane protein 3 (VAMP3) axis in paclitaxel (PTX) resistance and immune evasion in triple-negative breast cancer (TNBC).

**Methods:**

PTX resistance-related genes were screened by bioinformatics. CEBPD and VAMP3 expression in clinical TNBC samples was examined by immunohistochemistry. Three PTX-resistant TNBC cell lines (MDA-MB-231/PTX, MDA-MB-468/PTX and MDA-MB-453/PTX) were generated, and their drug resistance was analyzed. Autophagy of cells was analyzed by immunofluorescence staining. Interaction between CEBPD and VAMP3 promoter was identified by immunoprecipitation and luciferase assays. The extracellular expression of programmed cell death-ligand 1 (PD-L1) in TNBC cells was detected. Extracellular vesicles (EVs) from TNBC cells were isolated to examine their effects on CD8^+^ T cell exhaustion.

**Results:**

CEBPD and VAMP3 were upregulated in chemo-resistant tissue samples and in PTX-resistant TNBC cells. The CEBPD downregulation enhanced PTX sensitivity of cells. However, further upregulation of VAMP3 in cells restored PTX resistance, which was likely due to the activation of autophagy, as the autophagy antagonist chloroquine enhanced PTX sensitivity of cells. CEBPD was found to bind to the VAMP3 promoter to activate its transcription. The CEBPD/VAMP3 axis also increased the PD-L1 expression in the conditioned medium of TNBC cells. The TNBC cell-derived EVs increased the exhaustion of co-cultured CD8^+^ T cells.

**Conclusion:**

This study provides novel evidence that CEBPD plays a key role in enhancing PTX resistance in TNBC cells across various subtypes through VAMP3-mediated autophagy activation. Additionally, the CEBPD/VAMP3 axis also increases extracellular PD-L1 level, delivered by cancer cell-derived EVs, to suppress CD8^+^ T cell-mediated anti-tumor immune response. These significant observations may provide new insights into the treatment of TNBC, suggesting CEBPD and VAMP3 as promising targets to overcome treatment resistance.

**Supplementary Information:**

The online version contains supplementary material available at 10.1186/s13046-024-03041-8.

## Introduction

The 2020 global cancer statistics reveals female breast cancer as the most common cancer and the fifth leading cause of cancer mortality worldwide [1]. Triple-negative breast cancer (TNBC) is the most fatal subtype with several aggressive clinical characteristics such as higher invasiveness, higher metastatic and recurrent potentials, and shorter survival than other subtypes [2]. It constitutes roughly 15–20% of all breast cancer cases [3]. Owing to the negative expression of estrogen receptor, progesterone receptor, and human epidermal growth factor receptor-2, conventional therapies targeting these receptors are therefore ineffective for TNBC, leaving taxanes (such as paclitaxel; PTX)- and anthracyclines-based chemotherapy as the standard regimen [4, 5]. For patients with metastatic disease, chemotherapy has been suggested as the primary therapeutic option [6, 7]. In 2011, Lehmann and colleagues conducted gene expression profiling of tumor specimens from 587 TNBC patients, categorizing TNBC into six distinct subtypes: basal-like 1, basal-like 2, mesenchymal, mesenchymal stem-like, immunomodulatory, and luminal androgen receptor (LAR) [8]. Specifically, LAR TNBCs exhibit heightened resistance to both neoadjuvant and adjuvant chemotherapy, along with a notably poor pathological complete response [9]. Additionally, immunotherapy, mainly based on immune checkpoint inhibitors, has emerged as a promising strategy for TNBC [10]. Unfortunately, the development of drug resistance and the immunosuppressive tumor microenvironment in most patients largely limits the treatment efficacy and even leads to disease progression and death [11, 12]. Moreover, chemo drugs have been reported to increase the expression of programmed cell death-ligand 1 (PD-L1), a master negative regulator of T cell anti-tumor immunity, in breast cancer cells [13]. The causative factors for chemoresistance and the interaction between drug resistance and immune evasion remain elusive.

To identify the key molecular mechanisms related to chemoresistance, we investigated aberrantly expressed genes in chemo-resistant TNBC samples by analyzing two GEO datasets (GSE28784 and GSE90564; downloaded from https://www.ncbi.nlm.nih.gov/gds/) and consulting the human transcription factor database (TFDB; http://bioinfo.life.hust.edu.cn/HumanTFDB#!/download). We identified CCAAT enhancer binding protein delta (CEBPD), a member of the C/EBP transcription factor family that regulates inflammation and carcinogenesis [14], as a promising key factor that is aberrantly highly expressed TNBC samples. Indeed, studies suggest the causative correlation of CEBPD with chemoresistance and the consequent progression of urothelial carcinoma cells [15, 16]. This attracted our interests to probe its role in PTX resistance in TNBC. Moreover, through the bioinformatics analyses mentioned above, we predicted vesicle associated membrane protein 3 (VAMP3) as a target transcript of CEBPD. VAMP3 is a component of the soluble NSF attachment protein receptor (SNARE) complex and is required for the fusion between multivesicular bodies and autophagosomes to trigger autophagosome maturation [17]. Autophagy is a conserved catabolic process by which damaged cellular contents are taken up by autophagosomes and degraded, therefore alleviating stress [18]. It represents an important cell survival mechanism used by tumor cells to avoid cell death and generate treatment resistance [18, 19]. We therefore hypothesized that VAMP3 might have specific effects on chemoresistance in TNBC through its potential role in autophagy activation. Moreover, as its name suggests, VAMP3 has been closely linked to the vesicle fusion and exocytosis of extracellular vesicles (EVs) by interacting with its targeted SNARE counterparts [20, 21]. Considering the reported capability of chemotherapy drugs to induce PD-L1 expression, we wondered whether VAMP3 may induce extracellular PD-L1 and therefore affect anti-tumor response mediated by T cells. Taken together, this study aims to validate the interaction between CEBPD and VAMP3 and investigate their functions in PTX resistance and immunosuppression.

## Materials and methods

### Bioinformatics analysis

The PTX resistance-related genes were screened from the GEO databases GSE28784 and GSE90564, selecting significantly differentially expressed genes (DEGs) with *p* < 0.05. The list of human transcription factors was obtained from the HumanTFDB system (http://bioinfo.life.hust.edu.cn/HumanTFDB#!/download). The hTFtarget system (http://bioinfo.life.hust.edu.cn/hTFtarget/#!/) was then utilized to predict downstream target genes of the transcription factor CEBPD. The Jvenn system (https://jvenn.toulouse.inrae.fr/app/example.html) was used to generate a Venn diagram illustrating the intersections of different datasets. Kaplan–Meier Plotter (https://kmplot.com/analysis/) was employed to analyze the prognostic significance of gene expression on overall survival (OS) in breast cancer patients.

### Clinical samples

Post-chemotherapy biopsy tissue samples from 41 patients with TNBC were included for this research. Based on the therapeutic response of patients, the samples were divided into complete remission/partial remission (CR/PR) samples (*n* = 18; including 14 basal-like subtype, 3 mesenchymal subtype, and 1 LAR subtype) and stable disease/progressive disease (SD/PD) samples (*n* = 23; including 16 basal-like subtype, 4 mesenchymal subtype, and 3 LAR subtype). The two categories of patients showed no significant differences in terms of molecular subtypes (analyzed by Chi-square test). The usage of clinical samples was ratified by the Institute Review Board of the First Hospital of China Medical University. All procedures involving human samples were conducted in compliance with the *Declaration of Helsinki*.

### Immunohistochemistry (IHC)

Harvested tissue samples were fixed, paraffined, and cut into sections for IHC assay. According to the instruction of the kit (ab64261, Abcam Inc., Cambridge, MA, USA), the sections were successively dewaxed, deparaffined, rehydrated, treated with citric acid, blocked with H_2_O_2_ for 15 min, and treated with protein blockage reagent for 15 min. After that, the sections were probed by the antibodies against CEBPD (1:500, ab245214, Abcam) and VAMP3 (1:50, 10702–1-AP, Proteintech Group, Inc., Wuhan, Hubei, China) at 4℃ overnight, followed by incubation with biotinylated secondary antibody for 15 min and with streptavidin-peroxidase. After color development by DAB, the sections were counter-stained with hematoxylin. Additionally, the PD-L1 IHC 22C3 pharmDx kit (SKU: SK006, Agilent Technologies, Palo Alto, CA, USA) was utilized to assess the PD-L1 expression in tissue sections.

Protein expression in patient tissues was evaluated using IHC Profiler: An Open Source Plugin for the Quantitative Evaluation and Automated Scoring of Immunohistochemistry Images of Human Tissue Samples [22]. The final IHC score (0 ~ 3) of the sections was calculated as follows: percentage of high positive × 3 + percentage of positive × 2 + percentage of low positive × 1.

### Cell culture

Human TNBC cells MDA-MB-231 (HTB-26) and MDA-MB-468 (HTB-132) and CD8^+^ cytotoxic T cells (PCS-800–017) were procured from ATCC (Manassas, VA, USA). Another TNBC cell line, MDA-MB-453 (BNCC340797), was acquired from BeNa Culture Collection (Beijing, China). The TNBC cells were cultured in L-15 medium along with 10% fetal bovine serum (FBS) in a 37℃ incubator, and the CD8^+^ T cells were recovered and cultured in Roswell Park Memorial Institute (RPMI)-1640 medium containing 10% FBS.

### Construction of PTX-resistant TNBC cells

Approximately 2 × 10^6^ TNBC cells were incubated in culture flasks overnight and treated with 3 nM PTX. When the confluence reached 80%, the cells (2 × 10^6^) were digested, cultured in the flasks, and treated with PTX at higher concentrations. After five such repetitions, the PTX resistance of cells was evaluated by the CCK-8 assay. Those TNBC cells with an over twofold half maximal inhibitory concentration (IC50) of PTX than parental cells were designated as PTX-resistant cells.

### Cell counting kit-8 (CCK-8) assay for viability detection

Approximately 5,000 TNBC cells were resuspended in 90 µL culture medium and seeded in 96-well plates. On the next day, PTX reagent (HY-B0015, MedChemExpress, Monmouth Junction, NJ, USA) at an initial concentration of 20 µM was prepared, which was repeatedly diluted at a twofold gradient, with seven varying concentrations obtained. The TNBC cells were treated with PTX reagent at different concentrations for 48 h, followed by addition of 10 µL CCK-8 reagent (ab228554, Abcam) to each well. After 1 h of dark incubation at 37℃, the optical density (OD) value at 460 nm was examined. Cells treated with phosphate-buffered saline (PBS) instead of PTX were set to controls, whose OD460 value was defined as 100%, according to which the viability of PTX-treated cells was evaluated.

### Construction of cells with stable gene transfection

Short hairpin (sh) RNA of CEBPD (sh-CEBPD), overexpression plasmid of VAMP3 (oe-VAMP3), and the negative controls (NC; including sh-NC and oe-NC) used for lentivirus-based infection were procured from Genomeditech Co., Ltd. (Shanghai, China). In short, the TNBC cells were seeded in culture plates, and the lentivirus infection was performed when the confluence reached 60%. After 48 h, stably transfected cells were screened by the addition of corresponding antibiotics, and the successful gene expression alteration was confirmed by quantitative polymerase chain reaction (qPCR) and western blot (WB) analysis.

### Colony formation assay

The TNBC cells were treated with PTX for 48 h and harvested. Approximately 1,000 cells were cultured in six-well plates at 37℃ for two weeks. Thereafter, the cells were fixed with paraformaldehyde and stained with crystal violet (G1062, Solarbio Science & Technology Co., Ltd., Beijing, China) for 15 min. The formed cell colonies were counted under a microscope.

### Flow cytometry for cell apoptosis

According to the protocol of the Annexin V-fluorescein isothiocyanate (FITC)/propidium iodide (PI) kit (V13242, Thermo Fisher Scientific, Rockford, IL, USA), approximately 1 × 10^6^ PTX-treated TNBC cells were resuspended and seeded in 96-well plates, followed by dark incubation with FITC Annexin V and PI reagent for 15 min. Thereafter, the cells were washed and resuspended for apoptosis detection by a flow cytometer.

### Transwell assay for cell mobility

Transwell plates with or without Matrigel precoating were used to analyze invasion and migration of the PTX-treated TNBC cells, respectively. Approximately 5 × 10^4^ cells were resuspended in serum-free medium and seeded into the apical chambers, with serum-containing complete medium filled in basolateral chambers. After 24 h of incubation, the invasive or migratory TNBC cells were fixed, stained with crystal violet, and counted.

### Wound healing assay

TNBC cells were cultured in complete medium (containing 10% FBS) until confluency. Using a sterile 200 mL pipette tip, equidistant scratches were made on the cell monolayer, followed by capturing images. Subsequently, the cells were cultured for an additional 48 h in serum-free medium containing PTX. The migration of cells was observed, documented, and the migration rate over 48 h was calculated.

### RNA isolation and qPCR analysis

Total RNA from TNBC cells was isolated using the TRIzol reagent (Thermo Fisher Scientific) and used for cDNA synthesis using the PrimeScript RT Master Mix (RR036Q, Takara Holdings Inc., Kyoto, Japan). The qPCR analysis was performed following the protocol of TB Green® Premix Ex Taq II (RR820Q, Takara). Relative gene expression, with β-actin as the endogenous loading, was quantified by the 2^−ΔΔCt^ method. Below are the primer sequences: CEBPD (forward): 5ʹ-TCCGGCAGTTCTTCAAGCAGCT-3ʹ, (reverse): 5ʹ-GAGGTATGGGTCGTTGCTGAGT-3ʹ; VAMP3 (forward): 5ʹ-GCTCTCTGAGTTAGACGACCGT-3ʹ, (reverse): 5ʹ-CCAGAACAGTAATCCCGATTGCC-3ʹ; β-actin (forward): 5ʹ-CACCATTGGCAATGAGCGGTTC-3ʹ, (reverse): 5ʹ-AGGTCTTTGCGGATGTCCACGT-3ʹ.

### WB analysis

Total protein was isolated by RIPA buffer (Cat. No. 89900, Thermo Fisher Scientific), followed by protein concentration analysis using the bicinchoninic acid kit (Cat. No. 23225, Thermo Fisher Scientific). Total protein was separated by gel electrophoresis and wet-transferred to polyvinylidene fluoride membranes. The membranes were blocked by 5% non-fat milk, on which the proteins were probed by the antibodies of CEBPD (1:1,000, ab245214, Abcam), VAMP3 (1:500, 10702–1-AP, Proteintech Group, Inc., Wuhan, Hubei, China), and β-actin (1:1,000, #4970, Cell Signaling Technologies, Beverly, MA, USA) at 4℃ overnight, followed by incubation with biotinylated secondary antibody for 15 min. On the next day, the membranes were washed ad incubated with horseradish peroxidase-conjugated secondary antibody (1:5,000, ab6721, Abcam) at room temperature for 1 h. The blot bands were developed by enhanced chemiluminescence (D601039, Sangon Biotech Co., Ltd., Shanghai, China). Relative protein expression was then analyzed with β-actin as control as well.

### Chromatin immunoprecipitation (ChIP)-qPCR

As instructed by the Simple ChIP Enzymatic Chromatin IP Kit (9003, Cell Signaling Technology), the PTX-resistant TNBC cells were treated with formaldehyde for 15 min. The protein-chromatin crosslinking was terminated by glycine. The chromatin was digested by micrococcal nuclease and resuspended in ChIP buffer. Thereafter, the CEBPD antibody (1:30, ab245214, Abcam) or rabbit immunoglobulin G was added for IP reaction at 4℃ overnight. The chromatin was eluted and de-crosslinked, and the DNA was purified for qPCR analysis.

### Luciferase reporter assay

Binding site of CEBPD to the VAMP3 promoter was predicted from Jaspar (http://jaspar.genereg.net/). Wild-type (WT) VAMP3 or mutant-type (MUT) VAMP3 promoter sequence was inserted into pGL3-Basic luciferase reporter vectors. Human embryonic kidney (HEK) 293 T cells were seeded into 96-well plates. When the cell confluence reached 60%, they were transfected with sh-CEBPD along with the constructed reporter vectors using Lipofectamine 3000 (Thermo Fisher Scientific). After 48 h, the luciferase activity in cells was analyzed by the Dual Luciferase Reporter Gene Assay Kit (11402ES60, YEASEN Biotechnology Co. Ltd., Shanghai, China).

### Immunofluorescence staining

Approximately 5,000 PTX-treated cells were seeded in 24-well plates and fixed. After permeabilization with Triton-X-100 for 15 min, the cells were blocked by 5% goat serum for 1 h and incubated with the LC3B antibody (1:2,000, #3868, Cell Signaling Technology) at 4℃ overnight, followed by incubation with Alexa Fluor 488-conjugated secondary (#2975, Cell Signaling Technology) at room temperature for 40 min. The staining was analyzed under fluorescence microscopy.

### Isolation of extracellular vesicles (EVs)

Approximately 1 × 10^5^ TNBC cells were seeded in culture flasks and incubated at 37℃ for 24 h. The culture supernatant was collected, in which the EVs were isolated by differential ultracentrifugation. In short, the supernatant was centrifuged at 800 × *g* for 10 min, at 2,000 × *g* for 30 min, and then at 16,000 × *g* for 1 h to isolate the EVs, which were resuspended in PBS for subsequent analyses.

### *Co-culture of CD8* + *T cytotoxic cells and EVs*

The CD8^+^ T cells were activated by CD3^+^ and CD28^+^ stimulation. After that, 10 μg TNBC-derived EVs was co-cultured with 2 × 10^6^ activated CD8^+^ T cells for 48 h. The CD8^+^ T cells were then harvested, and the culture solution was centrifuged at 800 × g for 10 min to collect the supernatant for subsequent use.

### Enzyme-linked immunosorbent assay (ELISA)

Expression of PD-L1 in the culture supernatant of TNBC cells and the secretory cytokines in the co-culture system of CD8^+^ T cells and EVs was analyzed by ELISA. In short, the collected cell culture supernatant was added with the standard of each cytokine to the detection kit, followed by the addition of detection reagents. The OD450 value was detected using a microplate reader. Standard curves were produced according to the OD450 value of standards to evaluate the levels of cytokines of interests. Following ELISA kits were applied: PD-L1 (ab277712, Abcam), interferon gamma (IFN-γ; DY285B, R&D Systems, Minneapolis, MN, USA), Granzyme B (BMS2027-2, Thermo Fisher Scientific), and Perforin (BMS2306, Thermo Fisher Scientific).

### Xenograft mouse models

C57BL/6 J mice were purchased from Vital River Laboratory Animal Technology Co., Ltd. (Beijing, China) and used in protocols approved by the Animal Ethics Committee of the First Hospital of China Medical University. The mice were randomly allocated into four groups, *n* = 5 in each. Approximately 5 × 10^6^ MDA-MB-231/PTX cells were resuspended in 200 µL PBS and subcutaneously injected into mice. After one week, the growth of xenograft tumors was monitored. The major and minor axes of tumors were measured twice a week, on each Monday and Friday, respectively. The tumor volume (V) was calculated as follow: V = 1/2 × major axis × minor axis^2^. When the tumor volume reached 100 mm^3^, PTX treatment was applied at 20 mg/kg via intraperitoneal injection after every tumor volume measurement. Mice were euthanized by intraperitoneal injection of 1% pentobarbital sodium (150 mg/kg) on day 28, and tumor tissues were harvested and weighed.

### Xenograft tumor treatment

The tumor tissues were cut up and then soaked in RPMI-1640 medium containing 0.5 mg/mL collagenase IV, 0.1 mg/mL DNase I, 10% FBS, 1% antibiotics for 30 min, followed by centrifugation at 500 × *g* for 20 min to separate lymphocytes. The CD8^+^ T cells were then sorted using the Dynabeads FlowComp mouse CD8 kit (11462D, Thermo Fisher Scientific).

### Flow cytometry for T cell exhaustion analysis

The CD8^+^ cytotoxic T cells harvested from the co-cultured system and those from mouse xenograft tumors were subjected to flow cytometry to analyze T cell exhaustion. In short, the CD8^+^ T cells were resuspended in PBS and seeded in 96-well plates, followed by dark incubation with the antibodies of CD8a (1:100, 12–0081-82, Thermo Fisher Scientific) and Human Tim3 (1:20, 345022, Biolegend, San Diego, CA, USA)/Mouse Tim3 (1:100, MA5-17957, Thermo Fisher Scientific) for 30 min. Exhausted CD8^+^ T cells (Tim3^+^CD8^+^) were then screened using the flow cytometer.

### Statistical analysis

All data were analyzed by the Prism 8.0 software (GraphPad, La Jolla, CA, USA) and presented by the mean ± SD. Differences were compared using *t*-test. When more than two groups were compared, one- or two-way analysis of variance (ANOVA) were used with post-hoc tests using Tukey's or Sidak's multiple comparisons test. *P* < 0.05 is indicative of statistical significance. IC50 of PTX to cells was analyzed by nonlinear fitting analysis.

## Results

### CEBPD is highly expressed in PTX-resistant TNBC cells

**Fig. 1:**
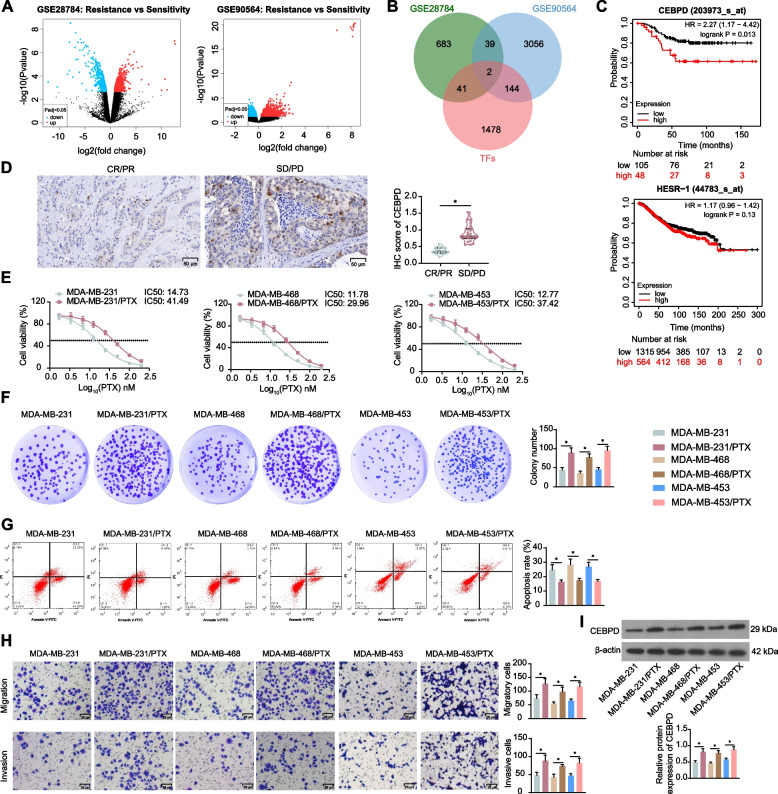
CEBPD is highly expressed in PTX-resistant TNBC cells. **A** volcano plots for DEGs identified using GSE28784 and GSE90564 datasets; **B** intersections between the two sets of DEGs and human transcription factors; **C** correlations between CEBPD and HEY1 (HESR-1) and OS of breast cancer analyzed by Kaplan–Meier Plotter analysis; **D** expression of CEBPD in biopsy tissues of SD/PD (*n* = 18) and CR/PR (*n* = 23) patients measured by IHC assay; **E** viability of parental (MDA-MB-231, MDA-MB-468, and MDA-MB-453) and PTX-resistant (MDA-MB-231/PTX, MDA-MB-468/PTX, and MDA-MB-453/PTX) cells and the PTX IC50 examined by CCK-8 assay (*n* = 3). Parental and PTX-resistant TNBC cells were treated with a fixed dose of 15 nM for 48 h for subsequent use. **F** colony formation ability of parental and PTX-resistant TNBC cells after PTX treatment (*n* = 3); **G** apoptosis of parental and PTX-resistant TNBC cells after PTX treatment determined by flow cytometry (*n* = 3); **H** migration and invasion of parental and PTX-resistant TNBC cells after PTX treatment determined by flow cytometry (*n* = 3); **I** CEBPD expression in parental and PTX-resistant TNBC cells measured by WB analysis (*n* = 3). Differences were analyzed by the *t*-test (**D**) or one-way ANOVA with Tukey's post-hoc test (**F**-**I**). IC50 of PTX in cells was analyzed by nonlinear fitting analysis (**E**). *p < 0.05

Two PTX resistance-related GEO datasets GSE28784 and GSE90564 were downloaded to analyze genes linking to PTX resistance in TNBC cell lines (Fig. 1A) (Supplementary file 1). Two sets of DEGs screened from the two datasets were intersected with the transcription factors predicted from the human TFDB, and two intersecting factors were identified obtained: CEBPD and HEY1 (Fig. 1B). Further analysis using Kaplan–Meier Plotter revealed that CEBPD has significant prognostic significance for the OS of breast cancer patients (*p* < 0.05), whereas HEY1 (HESR-1) showed no significant correlation with patient prognosis (Fig. 1C). Therefore, CEBPD was selected for further analysis. As introduced above that CEBPD has reportedly been linked to chemoresistance, we therefore focused on CEBPD to investigate its effect on PTX resistance. The IHC assay identified increased intensity of CEBPD staining in the biopsy tissues of SD/PD patients compared to that in tissues of CR/PR patients (Fig. 1D).

Three representative molecular subtypes of TNBC cells were selected for in vitro experiments, including basal-like subtype cells MDA-MB-468, mesenchymal subtype cells MDA-MB-231, and luminal androgen receptor subtype cells MDA-MB-453 [23–25]. The CCK-8 assay focusing on viability of parental and PTX-resistant (MDA-MB-231/PTX, MDA-MB-468/PTX, and MDA-MB-453/PTX) cells revealed that the drug resistant cell lines had significantly higher IC50 of PTX than the parental cell lines (Fig. 1E). Based on the CCK-8 results, PTX was applied at a dose of 15 nM for subsequent experiments. Not surprisingly, the PTX-resistant TNBC cell lines had conspicuously increased colony formation ability (Fig. 1F) and apoptosis resistance (Fig. 1G) following PTX treatment. Meanwhile, they showed higher migratory and invasive capacities than the parental cell lines in the Transwell assays (Fig. 1H). The increase in migration ability of PTX-resistant cells was further substantiated by the wound healing assay (Supplemental Fig S1A). Importantly, WB analysis identified elevated CEBPD expression in the PTX-resistant cell lines compared to the parental cell lines (Fig. 1I).

### Knockdown of CEBPD enhances PTX sensitivity of TNBC cells

**Fig. 2:**
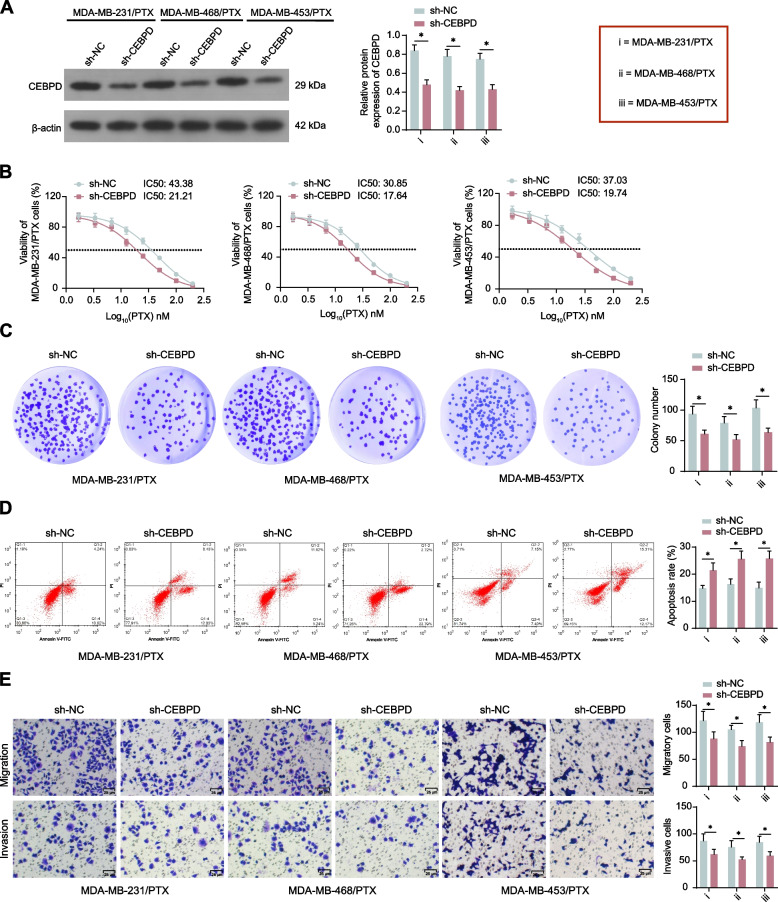
Knockdown of CEBPD enhances PTX sensitivity of TNBC cells. MDA-MB-231/PTX, MDA-MB-468/PTX, MDA-MB-453/PTX cells were administered lentivirus-carried sh-CEBPD or sh-NC. **A** CEBPD protein level in cells determined by WB analysis (*n* = 3); **B** viability of cells and the IC50 of PTX examined by CCK-8 assay (*n* = 3). Stably transfected cells were treated with 15 nM PTX for 48 h. **C** colony formation ability of cells analyzed by colony formation assay (*n* = 3); **D** apoptosis of cells determined by flow cytometry (*n* = 3); **E** migration and invasion of cells determined by Transwell assay (*n* = 3). Differences were analyzed by two-way ANOVA with Tukey's post-hoc test (**A**, **C**-**E**). IC50 of PTX to cells was analyzed by nonlinear fitting analysis (**B**). *p < 0.05

CEBPD downregulation was then induced in the PTX-resistant cells to investigate its exact role in PTX resistance. The stable PTX downregulation was confirmed by WB analysis (Fig. 2A). In this setting, the viability of cells under PTX treatment was reduced (Fig. 2B). Meanwhile, the CEBPD downregulation suppressed the colony formation (Fig. 2C), increased PTX-induced cell apoptosis (Fig. 2D), and significantly blocked the migration and invasion of the two PTX-resistant cell lines (Fig. 2E). Similarly, the wound healing assay also revealed that the migration of the PTX-resistant cells was decreased following CEBPD knockdown (Supplemental Fig S1B). This ample evidence indicates that the CEBPD indeed increases the resistance of TNBC cells to PTX.

### Knockdown of CEBPD suppresses VAMP3 transcription and cell autophagy

**Fig. 3:**
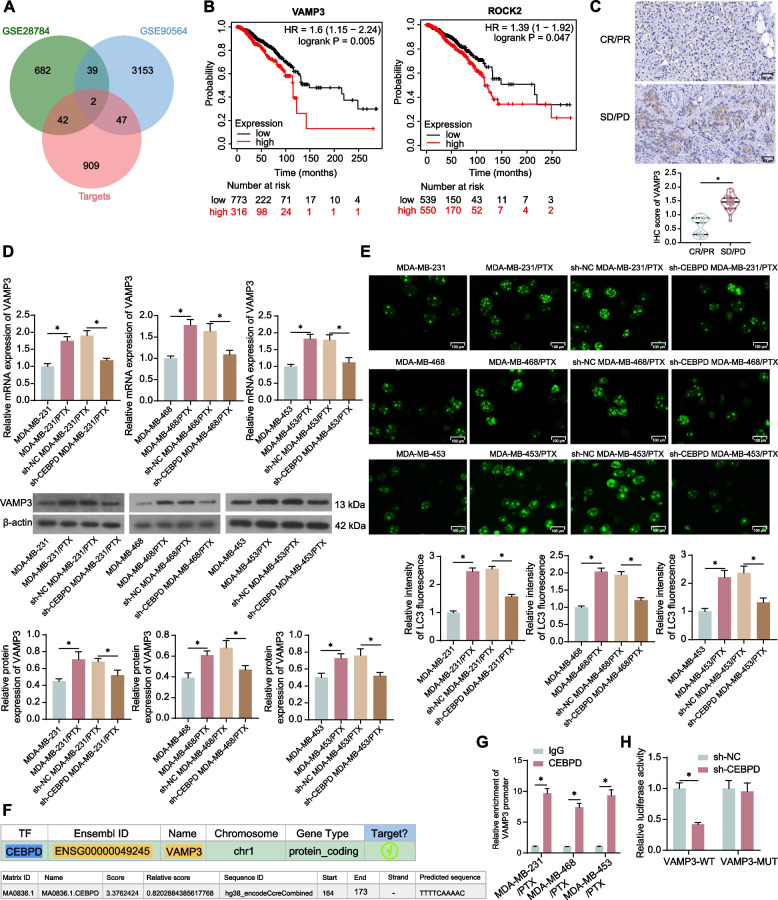
Knockdown of CEBPD suppresses VAMP3 transcription and cell autophagy. **A** intersections of top 1,000 candidate CEBPD targets with DEGs screened from GSE28784 and GSE90564 datasets; **B** correlations between CEBPD and HEY1 and OS of breast cancer analyzed by Kaplan–Meier Plotter analysis; **C** expression of VAMP3 in biopsy tissues of SD/PD (*n* = 18) and CR/PR (*n* = 23) patients measured by IHC assay. Parental or PTX-resistant TNBC cells were administered lentivirus-carried sh-CEBPD or sh-NC. **D** mRNA and protein levels of VAMP3 in cells detected by qPCR and WB analysis (*n* = 3); **E** autophagy activity in cells analyzed by immunofluorescence staining of LC3 (*n* = 3); **F** putative binding relationship and binding sites between CEBPD and VAMP3 promoter; **G** binding of CEBPD with VAMP3 promoter validated by ChIP-qPCR assay (*n* = 3); **H** regulatory role of CEBPD in VAMP3 transcription in 293 T cells with sh-NC or sh-CEBPD transfection determined by luciferase assay (**H**) (*n* = 3). Differences were analyzed by the *t*-test (C), one-way ANOVA followed by Tukey's multiple comparisons test (**D**-**E**) or by the two-way ANOVA followed by Sidak's post-hoc test (**G**-**H**). *p < 0.05

To probe the downstream molecules, we predicted the targets of CEBPD in hTFtarget system. The top 1,000 candidate targets were cross-referenced with those DEGs screened from GSE28784 and GSE90564 datasets, with ROCK2 and VAMP3 found to be intersected (Fig. 3A). Analysis results from Kaplan–Meier Plotter indicate that the expression of both VAMP3 and ROCK2 predicts worse OS in breast cancer patients. Among them, the prognostic significance of VAMP3 (ranked by *p*-value) is more pronounced. Therefore, VAMP3 was selected for further analysis (Fig. 3B). Another rationale for selecting VAMP3 as the study subject was its reported correlation with autophagosome formation and potential impact on chemoresistance as introduced above. The IHC assay also identified decreased VAMP3 expression in the tissues of CR/PR patients compared to that in SD/PD patients (Fig. 3C). Elevated mRNA and protein levels of VAMP3 were also detected in the MDA-MB-231/PTX, MDA-MB-468/PTX, and MDA-MB-453/PTX cell lines, and the sh-CEBPD administration in these cells led to a VAMP3 downregulation (Fig. 3D). Immunofluorescence staining showed that after PTX treatment, the PTX-resistant cells had increased intensity of LC3, a marker of autophagy activity, which was blocked upon CEBPD downregulation (Fig. 3E). We obtained the binding relationship and binding sites between CEBPD and VAMP3 promoter from the hTFtarget and JASPAR systems (Fig. 3F). The exact binding relationship was then validated in the MDA-MB-231/PTX, MDA-MB-468/PTX, and MDA-MB-453/PTX cell lines by the ChIP assay, where abundant VAMP3 promoter fragment was detected in the immune complexes precipitated by the CEPBD antibody (Fig. 3G). In the luciferase assay, downregulation of CEBPD in HEK293T cells blocked the luciferase activity of the VAMP3-WT reporter vector instead of the VAMP3-MUT reporter vector (Fig. 3H).

### VAMP3 overexpression induces autophagy and reduces PTX sensitivity of TNBC cells

**Fig. 4:**
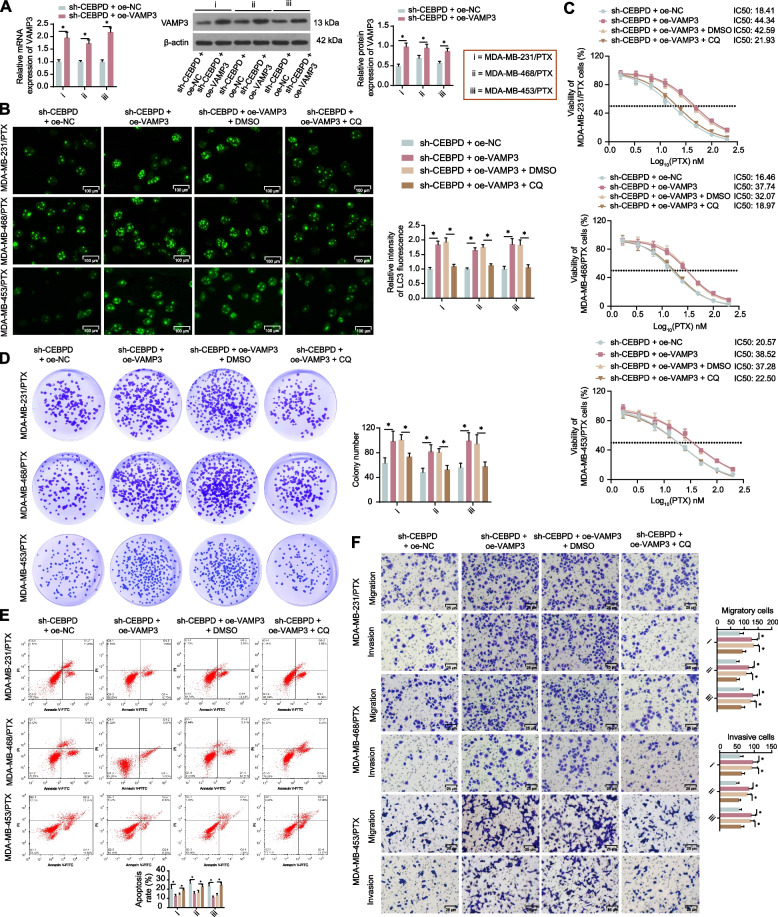
VAMP3 overexpression induces autophagy and reduces PTX sensitivity of TNBC cells. MDA-MB-231/PTX, MDA-MB-468/PTX, and MDA-MB-453/PTX cells stably transfected with sh-CEBPD were further administered oe-VAMP3. **A** mRNA and protein levels of VAMP3 in cells determined by qPCR and WB assays (*n* = 3). These cells were further treated with the autophagy antagonist CQ. **B** autophagy activity in cells analyzed by immunofluorescence staining of LC3; **C** viability of cells and the IC50 of PTX examined by CCK-8 assay (*n* = 3). These cells were additionally treated with 15 nM PTX for 48 h, **D** colony formation ability of cells analyzed by colony formation assay (*n* = 3); **E** apoptosis of cells determined by flow cytometry (*n* = 3); **F** migration and invasion of cells determined by Transwell assay (*n* = 3). Differences were analyzed by two-way ANOVA, followed by Tukey's (**B**, **D**-**F**) or Sidak's (**A**) post-hoc tests. IC50 of PTX to cells was analyzed by nonlinear fitting analysis (**C**). *p < 0.05

Gain-of-function studies of VAMP3 were then performed by administering lentivirus-carried oe-VAMP3 in the sh-CEBPD-treated PTX-resistant cells. The restoration of VAMP3 mRNA and protein levels was confirmed by qPCR and WB assays again (Fig. 4A). Moreover, the cells were treated with an autophagy antagonist chloroquine (CQ) to investigate the functions of VAMP3 and autophagy in the PTX resistance of cells. The immunofluorescence staining of LC3 showed that the VAMP3 increased the autophagy activity in cells, which was suppressed by CQ (Fig. 4B). CCK-8 assay revealed that the VAMP3 increased the IC50 of PTX in cells, which was reversed by CQ as well (Fig. 4C). The VAMP3 restoration in the two PTX-resistant cell lines also increased the colony formation (Fig. 4D), apoptosis resistance (Fig. 4E), as well as the migration and invasion (Fig. 4F) of cells under 15 nM PTX treatment. Of note, further administration of CQ in these cells blocked their malignant properties under PTX treatment (Fig. 4D-F). The wound healing assay also revealed that the migration ability of cells was promoted by VAMP3 overexpression but blocked by CQ treatment (Supplemental Fig S1C). These results indicate that VAMP3 increases autophagy activity in TNBC cells to enhance their PTX resistance.

### CEBPD/VAMP3 increases extracellular PD-L1 level in PTX-resistant TNBC cells

**Fig. 5:**
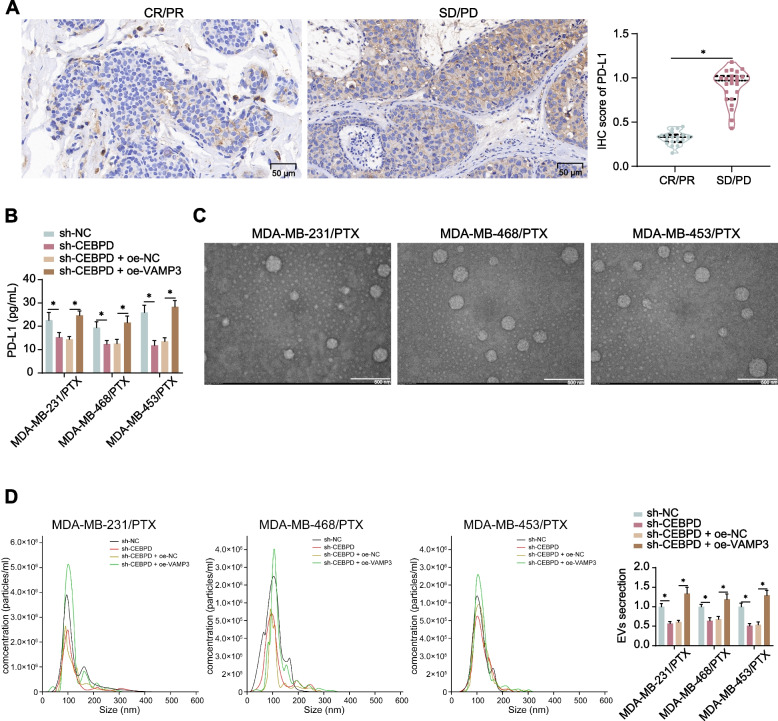
CEBPD/VAMP3 increases extracellular PD-L1 level in PTX-resistant TNBC cells. **A** expression of PD-L1 in biopsy tissues of SD/PD (*n* = 18) and CR/PR (*n* = 23) patients measured by IHC assay; **B** concentration of PD-L1 in the culture supernatant of sh-CEBPD- or sh-CEBPD + oe-VAMP3-treated PTX-resistant TNBC cells detected by ELISA kit (*n* = 3); **C** morphology of the isolated EVs under transmission electron microscopy; **D** particle size distribution and concentration of the isolated EVs determined by nanoparticle tracking analysis (*n* = 3). Differences were analyzed by the *t*-test (**A**) or two-way ANOVA with Tukey's post-hoc test (**B**, **D**). *p < 0.05

Chemoresistance has been reported to induce the PD-L1 expression and consequently confer cancer cells the capacity to evade from cytotoxic T cells-mediated immune response [26]. We therefore detected the PD-L1 expression in the collected tissues. Of note, elevated PD-L1 expression was detected in the CR/PR patients compared to the SD/PD groups (Fig. 5A). In the culture medium of MDA-MB-231/PTX, MDA-MB-468/PTX, and MDA-MB-453/PTX, the concentration of PD-L1 was decreased by CEBPD downregulation but increased by VAMP3 restoration (Fig. 5B). As the term suggests, VAMP3 is a vesicle-associated membrane protein and closely linked to the vesicle fusion and exocytosis of EVs. We therefore further isolated EVs from the TNBC cells, which presented typical cup-shaped under transmission electron microscopy (Fig. 5C). The nanoparticle tracking analysis showed that the particle size of EVs was mainly distributed in ~ 120 nm, and the EVs concentration was reduced by CEBPD downregulation and increased after VAMP3 restoration (Fig. 5D).

### *CEBPD/VAMP3 affects the cytotoxicity of CD8*^+^*T cells*

**Fig. 6:**
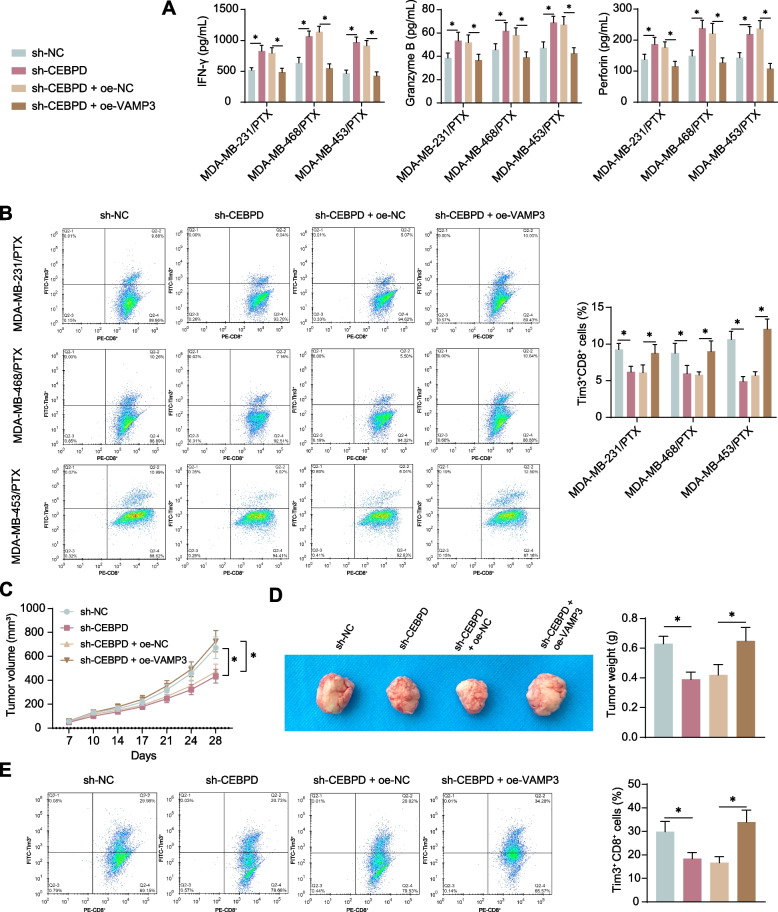
CEBPD/VAMP3 affects the cytotoxicity of CD8^+^ T cells to the PTX-resistant TNBC cells. EVs derived from differentially treated (sh-NC, sh-CEBPD, sh-CEBPD + oe-NC, and sh-CEBPD + oe-VAMP3) PTX-resistant cells were collected, which were co-cultured with activated CD8^+^ T cells for 48 h. **A**, concentrations of IFN-γ, Granzyme B, and Perforin in the supernatant of co-culture system examined using ELISA kits (*n* = 3); **B**, proportion of exhausted CD8^+^ T cells (Tim3^+^CD8^+^) analyzed by flow cytometry (*n* = 3). Differentially treated MDA-MB-231/PTX cells were injected into C57BL/6 J mice. When the tumor volume reached 100 mm^3^, PTX treatment was applied at 20 mg/kg via intraperitoneal injection twice a week. **C**, growth rate of xenograft tumors in mice (*n* = 5); **D**, weight of xenograft tumors on day 28 (*n* = 5); **E**, proportion of exhausted CD8^+^ T cells (Tim3^+^CD8.^+^) in the tumor-infiltrating lymphocytes analyzed by flow cytometry (*n* = 5). Differences were analyzed by the one-way (**D**-**E**) or two-way (**A**-**C**) ANOVA with Tukey's post-hoc test (**B**, **D**). *p < 0.05

The exact role of the CEBPD/VAMP3 axis in immune response activity was then analyzed in vitro. In the supernatant of co-culture system of CD8^+^ T cells and TNBC cell-derived EVs, the concentrations of IFN-γ, Granzyme B, and Perforin were increased in the CEBPD silencing condition but decreased after further oe-VAMP3 treatment in TNBC cells (Fig. 6A). The CD8^+^ T cells were harvested after co-culture, in which the number of exhausted T cells was reduced in the setting of CEBPD knockdown but increased upon VAMP3 overexpression (Fig. 6B). Moreover, MDA-MB-231/PTX cells were injected into mice to induce subcutaneous xenograft tumors, followed by PTX treatment. Administration of sh-CEBPD in cells reduced the volume and weight of the formed tumors; however, further overexpression of VAMP3 in cells led to an increase in the volume and weight of tumors (Fig. 6C-D). Similarly, the CD8^+^ T cells were sorted from the harvested xenograft tumor tissues, in which the cell exhaustion was decreased by sh-CEBPD but promoted by oe-VAMP3 (Fig. 6E).

## Discussion

The chemoresistance has long been a major obstacle in anti-cancer treatment and increasingly coming to the fore in cancer management researches, and its interaction with immunosuppression is rather interesting. In this study, we confirm CEBPD as a chemoresistance-related factor and VAMP3 as its important physiologic substrate implicated in PTX resistance and elevated PD-L1 expression.

Given that GEO datasets are convenient tools for the identification or validation of differentially expressed molecules in pathological conditions including drug resistance [27, 28], we analyzed GSE28784 and GSE90564 datasets and identified CEBPD as a significantly highly expressed transcription factor in chemo-resistant samples. Studies have indicated the pro-survival of CEBPD in several human malignancies. For instance, it mediated glioblastoma survival through catalase-mediated hydrogen peroxide clearance [29]. In gallbladder cancer, CEBPD activated myeloid cell leukemia 1 transcription to prevent tumor cells from stress-induced death [30]. Moreover, it has been reported to trigger angiogenesis to promote urothelial carcinoma progression [31]. In breast cancer, CEBPD reportedly links to interleukin-6 and hypoxia-inducible factor 1 signaling to promote cancer stem cell-associated phenotypes [32], which is a well-known factor correlated with drug resistance [33]. Indeed, CEBPD has been reported to be associated with chemoresistance and the consequent progression of urothelial carcinoma cells [15, 16]. However, the exact role of CEBPD in PTX resistance in TNBC remains unknown. In this study, we first validated increased CEBPD expression in chemo-resistant tissue samples and in PTX-resistant TNBC cells despite the molecular subtypes (basal-like type, mesenchymal type, and LAR type). Importantly, CEBPD downregulation enhanced PTX sensitivity in all three types of TBNC cells, as manifested by decreased IC50 of PTX, increased cell apoptosis while suppressed cell migration and invasion. This evidence elucidates the direct correlation between CEBPD upregulation and PTX resistance in TNBC, including in the reportedly less chemotherapy responsive LAR type [9].

Our further molecular investigation identified VAMP3 as a downstream substrate of CEBPD, which was validated by luciferase and immunoprecipitations assays, and elevated VAMP3 was detected in drug-resistant tissue and cells. The v-SNARE protein VAMP3 reportedly mediates heterotypic fusions between ATG9- and ATG16L1-containing vesicles in recycling endosomes, which has a causative effect on the autophagosome genesis [34]. The autophagy is indeed a key mechanism by tumor cells to survive under stress including chemotherapy, and therefore representing a key target for cancer treatment [18, 35]. Interestingly, VAMP3 has also been found to suppresses caspase-dependent apoptosis to enhance chemoresistance [36]. We observed that VAMP3 overexpression in the TNBC cells restored PTX resistance suppressed by CEBPD downregulation, along with the increased immunostaining of LC3. Given the fact that CQ treatment enhanced the PTX sensitivity of cells, we considered that VAMP3-mediated PTX resistance was due in part to the activation of autophagy. Chemo drugs have been reported to increase the expression of PD-L1 in cancer cells and in the tumor microenvironment [13, 37]. Several drug-resistant tumor cells have been found to exhibit elevated PD-L1 expression, reportedly due to the activation of the JNK/c-Jun signaling pathway [26]. Moreover, EVs derived from tumor cells can secret PD-L1, which binds to its receptor programed cell death 1 on the surface of activated T cells, therefore activating immune checkpoint signaling and inducing T cell dysfunction [38]. As VAMP3 is a ubiquitously expressed v-SNARE protein that leads to vesicle fusion and exocytosis by interacting with its targeted SNARE counterpart [20, 21], we wondered whether the enhanced VAMP3 expression in PTX-resistant cells might increase EVs secretion and therefore enhance the extracellular PD-L1 level. Indeed, we confirmed that the concentration of EVs and the extracellular PD-L1 level in culture medium of PTX-resistant TNBC cells were decreased by CEBPD silencing but increased upon VAMP3 restoration. In the VAMP3 overexpressing condition, EVs increased the exhaustion of CD8^+^ T cells and reduced their cytotoxicity. Therefore, it can be inferred that VAMP3 can increase the extracellular PD-L1 expression to suppress the T cell-mediated anti-tumor immune response.

## Conclusions

**Fig. 7:**
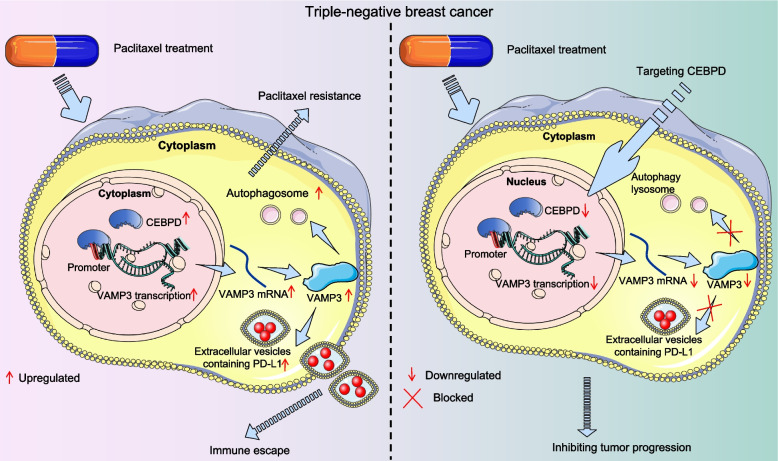
Graphical abstract. In PTX-resistant TNBC cells, elevated CEBPD expression promotes transcription and expression of VAMP3, which increases formation of autophagosomes to aggravate chemoresistance. Moreover, VAMP3 increases the secretion of PD-L1 to induce immunosuppression

In summary, our study provides novel evidence implicating CEBPD in treatment resistance in TNBC through transcriptional activation of VAMP3. First, it enhances PTX resistance in TNBC cells across various subtypes by increasing VAMP3-mediated autophagy activation. Additionally, the CEBPD/ VAMP3 axis increases extracellular PD-L1 levels via the paracrine EVs, consequently dampening the anti-tumor immune response (Fig. 7). Targeting any component in this axis could potentially enhance the PTX sensitivity in TNBC cells and bolster anti-tumor immune response. However, it's crucial to acknowledge the limitation of the study, notably the small sample size of available clinical TNBC patient cohorts, which may have influenced the observed lack of major differences in molecular subtypes between drug-resistant and drug-sensitive cohorts. Moving forward, we aim to explore the relationship between the CEBPD/VAMP3 axis and PTX resistance across various TNBC subtypes and in larger clinical cohorts to further validate our findings. Additionally, while we have demonstrated that knockdown of CEBPD could heighten PTX sensitivity in developed treatment-resistant cells, exploring whether the overexpression of CEBPD or VAMP3 would confer PTX resistance to parental (wild-type) TNBC cells could offer further insights. Regrettably, such additional experiments were not conducted primarily due to time and funding constraints. We aspire to address these gaps in our future research endeavors.

### Supplementary Information

**Supplementary Material 1.**

**Supplementary Material 2.**

## Data Availability

The data that support the findings of this study are available from the corresponding author upon reasonable request.

## Abbreviations

CEBPD: CCAAT enhancer binding protein delta
CQ: Chloroquine
CR/PR: Complete remission/partial remission
EVs: Extracellular vesicles
FITC: Fluorescein isothiocyanate
IC50: Half maximal inhibitory concentration
IFN-γ: Interferon gamma
IHC: Immunohistochemistry
oe-: Overexpression plasmid
PD-L1: Programmed cell death-ligand 1
PI: Propidium iodide
PTX: Paclitaxel
SANRE: Soluble NSF attachment protein receptor
SD/PD: Stable disease/progressive disease
TFBD: Human transcription factor database
TNBC: Triple-negative breast cancer
VAMP3: Vesicle associated membrane protein 3
