# Supplementary material for: CCAAT enhancer binding protein delta activates vesicle associated membrane protein 3 transcription to enhance chemoresistance and extracellular PD-L1 expression in triple-negative breast cancer
Source: J Exp Clin Cancer Res. 2024 Apr 16;43:115. doi: 10.1186/s13046-024-03041-8 (PMC11020785; doi:10.1186/s13046-024-03041-8)
Supplement: Supplementary file 2 — Supplementary Material 2. [file 13046_2024_3041_MOESM2_ESM.docx]

**Supplemental Fig S1**

**
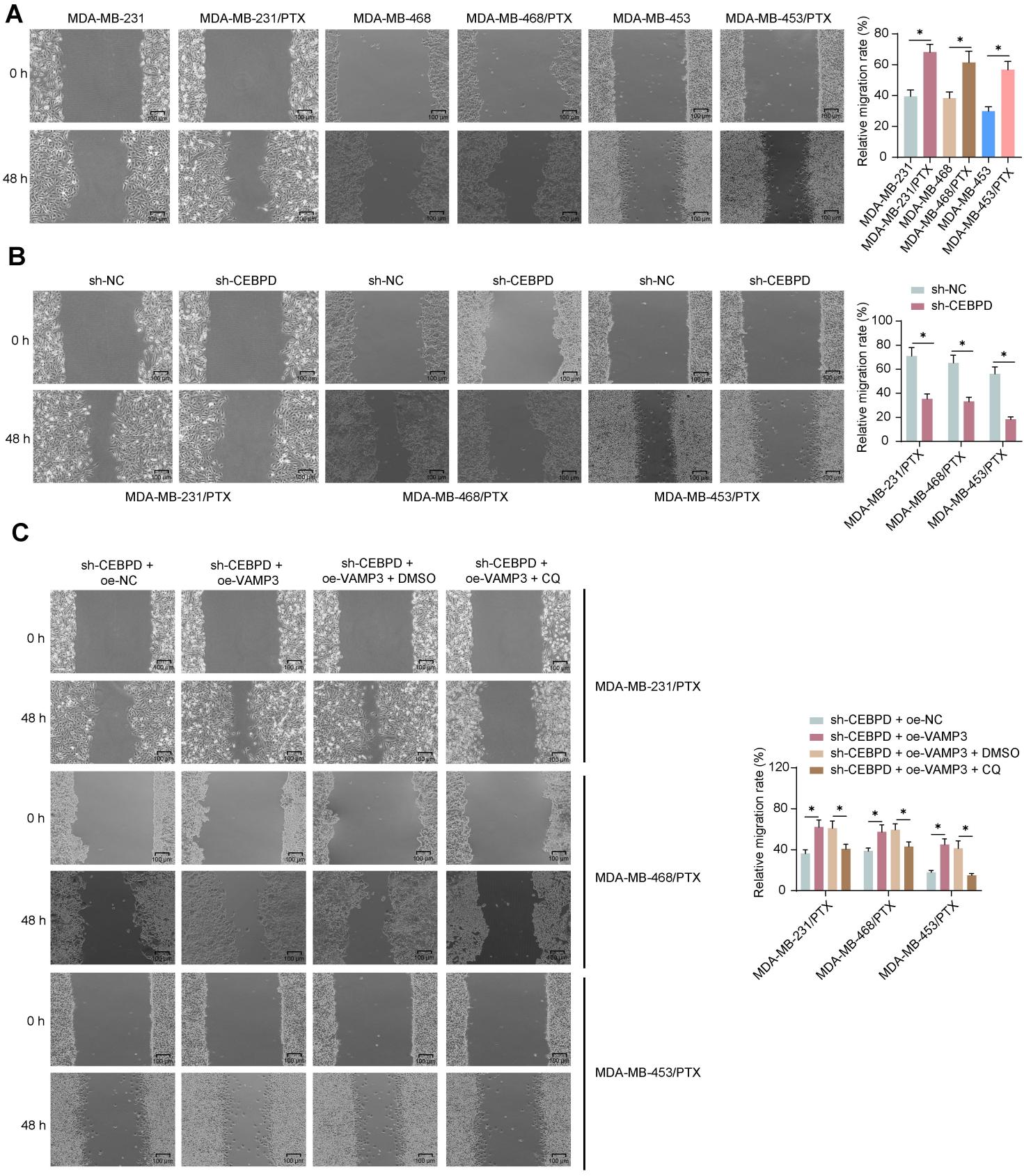
**

**Supplemental Fig S1** Wound healing assay for migration of TNBC cells. A, migration ability of parental (MDA-MB-231, MDA-MB-468, MDA-MB-453) and PTX-resistant (MDA-MB-231/PTX, MDA-MB-468/PTX, and MDA-MB-453/PTX) cells after 15 nM PTX treatment; B, migration ability of PTX-resistant cells upon CEBPD knockdown and 15 nM PTX treatment; C, migration ability of PTX-resistant cells upon sh-CEBPD and oe-VAMP3 transfection and DQ or DMSO treatment. Differences were compared by the one-way (A) or two-way (B-C) ANOVA, followed by Tukey's (A and C) or Sidak's post-hoc tests (B). **p* < 0.05.
